# Supplementary material for: The Construction and Immunoadjuvant Activities of the Oral Interleukin-17B Expressed by Lactobacillus plantarum NC8 Strain in the Infectious Bronchitis Virus Vaccination of Chickens
Source: Vaccines (Basel). 2020 Jun 6;8(2):282. doi: 10.3390/vaccines8020282 (PMC7350006; doi:10.3390/vaccines8020282)
Supplement: Supplementary file 1 [file vaccines-08-00282-s001.pdf]

Supplementary Materials

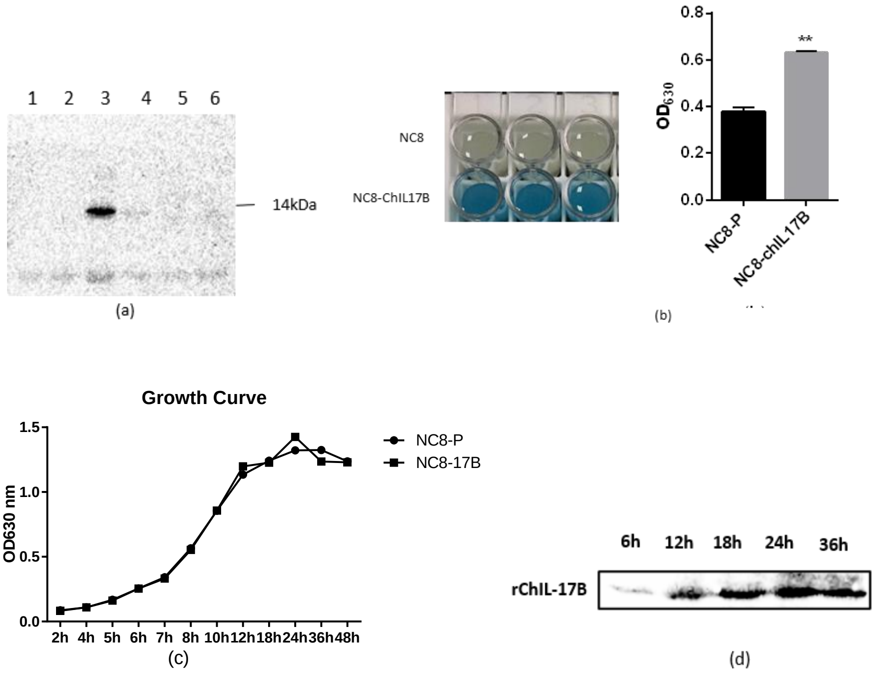

Figure S1. Analysis of rChIL-17B expression in recombinant *Lactobacillus plantarum* NC8-ChIL17B cells. (**a**,**b**) Expression pattern of rChIL-17B detected by Western blot and whole-cell ELISA. (**a**) Lanes 1–6 represent the protein in the culture supernatant, the intracellular protein, and the cell surface protein of the NC8-ChIL17B strain (Lanes 1–3) and the NC8-P strain (Lanes 4–6), respectively. The proteins in the culture supernatant were extracted with 100% trichloroacetic acid (TCA) (w/v) solution and then condensed from 50–1 mL. The cell surface proteins were extracted with 2% sodium dodecyl sulfate (SDS) and 1% mercaptoethanol at 70 °C, while the intracellular proteins were extracted first with SDS and 1% mercaptoethanol at 70 °C and then with 6 mg/mL lysozyme. (**b**) The results of the whole-cell ELISA. The NC8- ChIL17B culture was collected by centrifugation at 4000 *g* for 5 min, and then washed twice using sterile PBS. The pellet was then resuspended and fixed with 4% paraformaldehyde (Biosharp life science, Hefei, China). The mouse anti-His-Tag monoclonal antibody (Abcam Ltd, Shanghai, China) was used as the primary antibody, and HRP-labeled goat anti-mouse IgG (H + L) (Proteintech Group, Inc., Wuhan, China) was used as the secondary antibody. The picture on the left shows the color change after adding TMB chromogenic substrate. The picture on the right shows the absorbance of the whole-cell ELISA. The value of NC8- ChIL17B was very significantly higher than in the NC8-P control. (**c**,**d**) Detection of the best harvest time of the NC8-ChIL17B strain by growth curve measurement and Western blot. (**c**) The growth curves of NC8-ChIL17B and NC8-P. (**d**) The expression of rChIL17B at 6 h, 12 h, 18 h, 24 h, and 36 h. The optimal harvest time of NC8-ChIL17B was 24 h after culture, when the absorbance reached around 1.42, detected under 630 nm wavelength. ** $p < 0.01$.

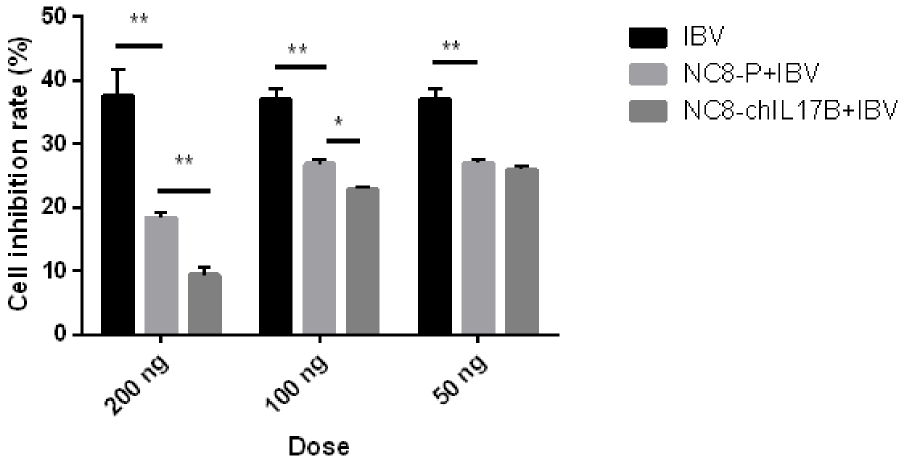

Figure S2. Detection of the cell activity inhibition rate by the MTT Cell Proliferation and Cytotoxicity Assay kit. The HD11 cells were cultured in a 96 well plate and treated with NC8-P, NC8-ChIL17B, or PBS, along with 2 MOI infectious bronchitis virus (IBV). Cell wells with PBS only were used as the healthy cell controls. After incubating for 24 h, the cells were detected using the MTT Cell Proliferation and Cytotoxicity Assay Kit (Beyotime Biotechnology Ltd, Shanghai, China) according to the manufacturer's instructions. The percentage of inhibition of the cell activity (%) = (PBS cell control group OD_{450} − treated group OD_{450}) / (PBS cell control group OD_{450} − blank well OD_{450}) × 100. The lower the percentage, the better the cell activity. Here, the NC8-ChIL17B-treated group had the lowest cell activity inhibition rate compared to the positive control IBV group ($p < 0.01$) and the NC8-P control group ($p < 0.05$) under the dose of 50 ng/mL. Tukey's multiple comparison test in GraphPad Prism 6 (GraphPad Software Inc., La Jolla, CA, USA) was used for statistical analysis. * $p < 0.05$, ** $p < 0.01$.

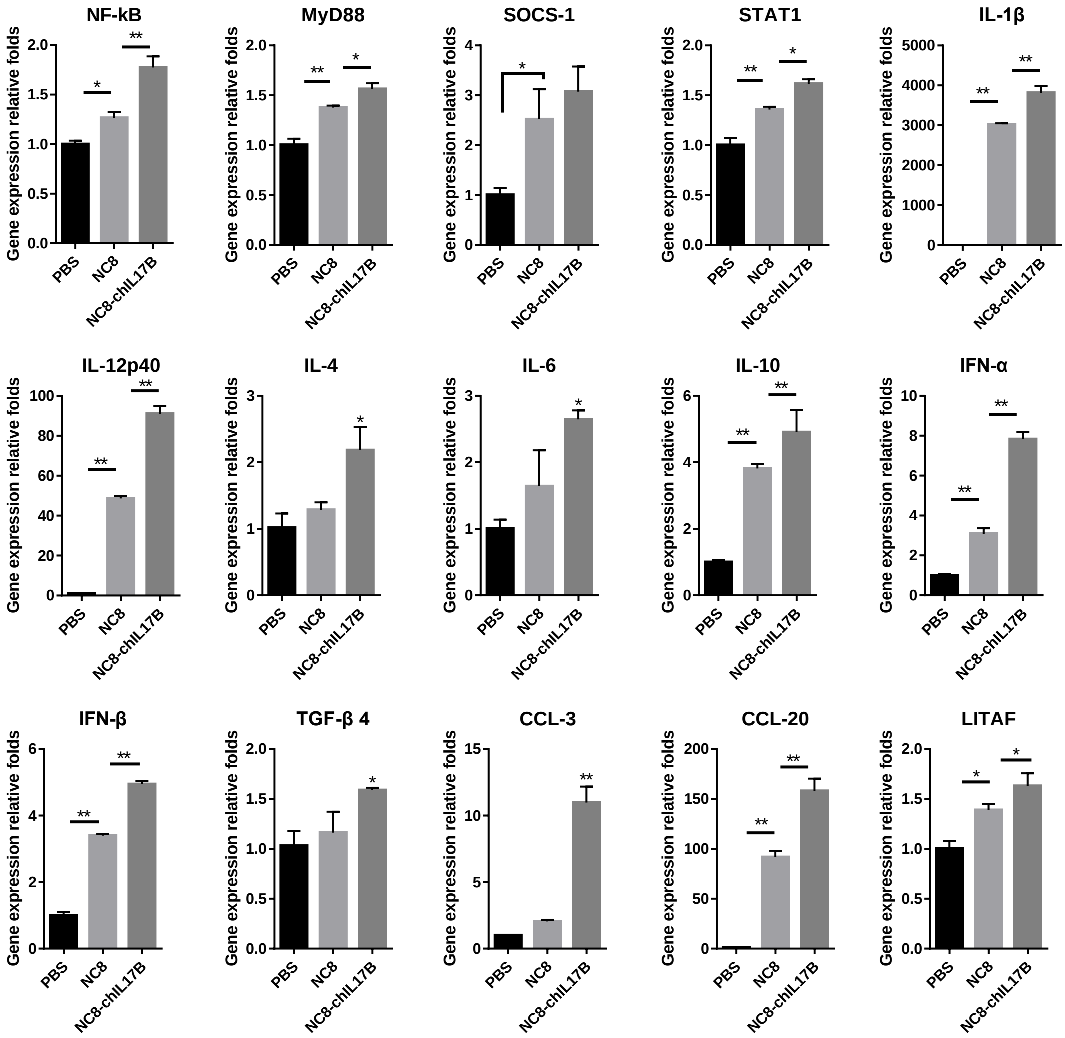

Figure S3. The expression levels of the immune-related genes of HD11 cells treated with recombinant *Lactobacillus plantarum* NC8-ChIL17B protein. The primers used in the qRT-PCR are shown in Supplementary Table S1. The expression levels of NF-κB, MyD88, SOCS1, STAT1, IL-1β,IL-12P40, IL-4, IL-6, IL-10, INF-α, IFN-β, TGF-β4, CCL-3, CCL-20, and LITAF in rChIL-17B-stimulated HD11 cells were significantly upregulated compared to the PBS and NC8-P controls (p < 0.05 or p < 0.01). Statistical analysis of the data was performed via ordinary one-way ANOVA Tukey's multiple comparison test using GraphPad Prism 6 (GraphPad Software Inc., La Jolla, CA, USA). * p < 0.05, ** p < 0.01.

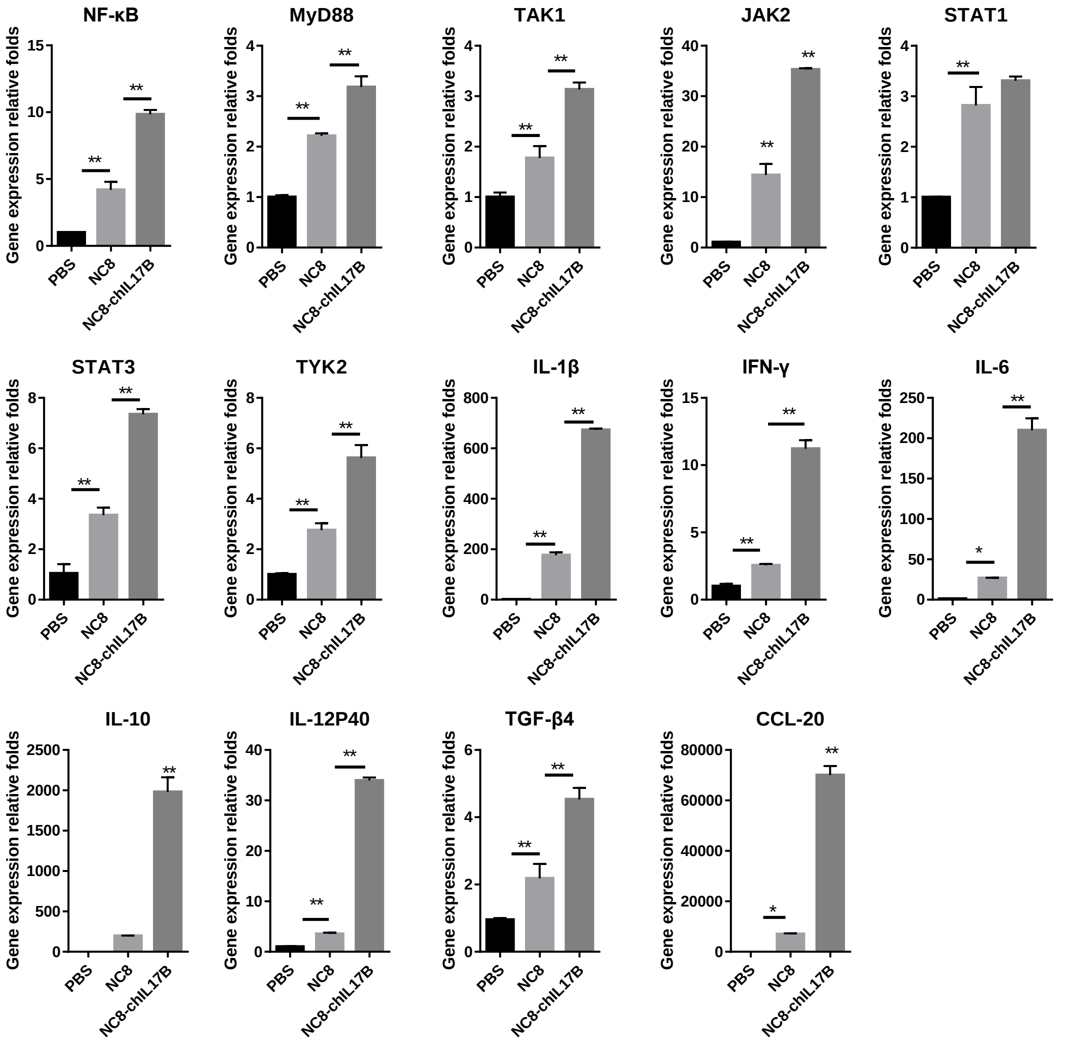

Figure S4. The expression levels of the immune-related genes of fibroblast cell line DF-1 cells treated with recombinant *Lactobacillus plantarum* NC8-ChIL17B protein. The primers used in the qRT-PCR are shown in Supplementary Table S1. The expression levels of NF-κB, MyD88, TAK1, JAK2, STAT1, STAT3, TYK2, IL-1β, IL-4, IL-6, IL-10, IFN-γ, TGF-β4, and CCL-20 in rChIL-17B-stimulated DF-1 cells were significantly upregulated compared to the PBS and NC8-P controls. Statistical analysis of the data was performed by the ordinary one-way ANOVA Tukey's multiple comparison test using GraphPad Prism 6 (GraphPad Software Inc., La Jolla, CA, USA). * $p < 0.05$, ** $p < 0.01$.

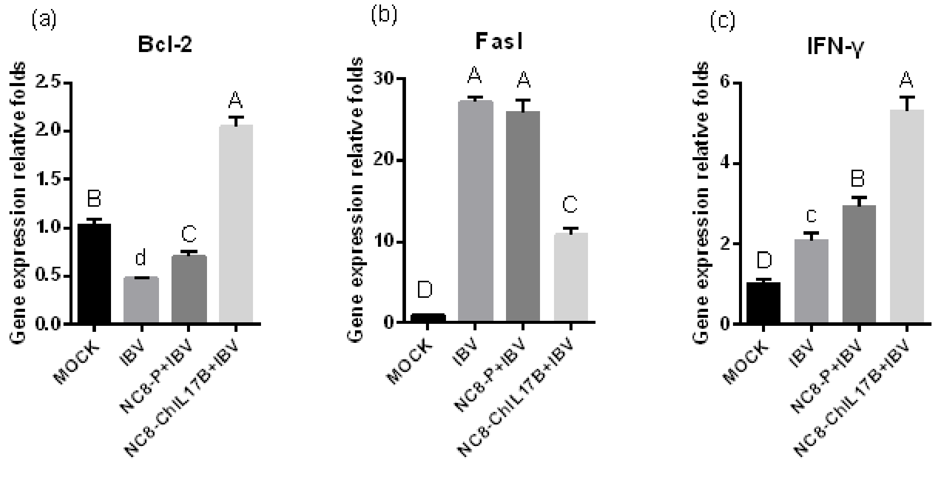

Figure S5. Changes in the mRNA levels of Bcl-2 (**a**), FasL (**b**), and interferon gamma IFN-γ (**c**) genes in IBV-infected HD11 cells treated with a NC8-ChIL17B mixture solution. Bcl-2 is an important apoptosis-suppression gene. The FasL gene participates in the induction of cell apoptosis. The significant upregulation of the Bcl-2 gene and the downregulation of the FasL gene indicated that the rChIL-17B could, through inhibiting the apoptosis of IBV-infected cells, inhibit IBV replication. Additionally, IFN-γ, an antiviral cytokine, was also upregulated in IBV-infected cells, and the rChIL-17B-treated cells showed higher mRNA levels compared to the IBV and the NC8-P+IBV controls. Different letters indicate significant differences: Capital letters indicate a significant difference at $p < 0.01$, and lower-case letters indicate a significant difference at $p < 0.05$.

Table S1. The primers used for quantitative real-time polymerase chain reaction (qRT-PCR) to detect the gene expression in HD11 and DF-1 cells.

Primers	F/R	Oligonucleotide Sequences (5′ - 3′)	GenBank Accession No.
GAPDH	F	TGCTGCCCAGAACATCATCC	NM_204305
GAPDH	R	ACGGCAGGTCAGGTCAACAA	-
STAT1	F	TTGTAACTTCGCTATTGGTATTCC	NM_001012914
STAT1	R	TTCCGTGATGTGTCTTCCTTC	-
STAT3	F	AGGGCCAGGTGTGAACTACT	NM_001030931
STAT3	R	CCAGCCAGACCCAGAAAG	-
IL-1β	F	TCGGGTTGGTTGGTGATG	NM_204524
IL-1β	R	TGGGCATCAAGGGCTACA	-
TGF-β4	F	CGTGCCCGTACATCTGGAG	AF459839.1
TGF-β4	R	GAGGGGGTCGAGGGTCTG	-
JAK2	F	CAGATTTCAGGCCGTCATTT	NM_001030538
JAK2	R	ATCCAAGAGCTCCAGTTCGTAT	-
IL-6	F	CAAGGTGACGGAGGAGGAC	JQ897539
IL-6	R	TGGCGAGGAGGGATTTCT	-
IFN-γ	F	AGCTGACGACGGTGGACCTATTATT	HQ739082
IFN-γ	R	GGCTTTGCGCTGGATTC	-
IL-4	F	AGCACTGCCACAAGAACCTG	NM_001007079.1
IL-4	R	CCTGCTGCCGTGGGACAT	-
IL-12p40	F	AGATGCTGGCAACTACACCTG	NM_213571
IL-12p40	R	CATTTGCCCATTGGAGTCTAC	-
IL-10	F	CTGTCACCGCTTCTTCACCT	AJ621254
IL-10	R	ACTCCCCCATGGCTTTGTA	-
CCL-20	F	AGGCAGCGAAGGAGCAC	NM_204438
CCL-20	R	GCAGAGAAGCCAAAATCAAAC	-
CCL-3	F	CATTGCCTCCGCCTACAT	EU999777
CCL-3	R	ACTCCTCGGGGTTTACACATA	-
IFN-α	F	AACCACCCACGACATCCTTC	NM_205427
IFN-α	R	CAAGCATTGCTCGAGGTGC	-
IFN-β	F	CTTGCCCACAACAAGACGTG	NM_001024836
IFN-β	R	GTGTTTTGGAGTGTGTGGGC	-
NF-κB	F	AGAAAAGCTGGGTCTTGGCA	NM_205134
NF-κB	R	CCATCTGTGTCAAAGCAGCG	-
MyD88	F	GGTTCTGGACAAGACTG	NM_001030962
MyD88	R	ATGCTGTAGGAACACCGT	-
SOCS1	F	CTACTGGGGACCGCTGACC	NM_001137648
SOCS1	R	TTAACACTGATGGCAAAGAAACAA	-
TAK1	F	CCAGGAAACGGACAGCAGAG	XM_015284677
TAK1	R	GGTTGGTCCCGAGGTAGTGA	-
LITAF	F	TGTGTATGTGCAGCAACCCGTAGT	AY765397
LITAF	R	GGCATTGCAATTTGGACAGAAGT	-

Note: F—forward; R—reverse; GAPDH—glyceraldehyde-3-phosphate dehydrogenase; STAT—signal transducer and activator of transcription; IL—interleukin; JAK—Janus kinase; TGF—transforming growth factor; IFN—interferon; NF-κB—nuclear factor kappa-B; MyD88—myeloid

differentiation factor 88; SOCS—suppressor of cytokine signaling; TAK—transforming growth factor kinase; LITAF—LPS-induced TNF-α.
